# Traumatic Dislocation of Tibialis Posterior Tendon: A Case Report and Literature Review

**DOI:** 10.7759/cureus.10885

**Published:** 2020-10-10

**Authors:** Wejdan M Alamri, Razan Aljeaan, Ahmed K Almulhim, Hosny Saleh

**Affiliations:** 1 Orthopedic Surgery, King Fahad Hofuf Hospital, Al-Ahsa, SAU; 2 Orthopedic Surgery, King Fahad Medical Military Complex, Dhahran, SAU

**Keywords:** tibialis posterior tendon dislocation, trauma, ankle sprain

## Abstract

Traumatic dislocation of the tibialis posterior tendon is one of the significantly rare conditions that we might deal with in the emergency department. Approximately only 50 cases have been reported in the literature, and, usually, this condition is misdiagnosed as an ankle sprain in acute settings. It might be neglected in case of improper clinical examination and imaging techniques.

We present a case of a 39-year-old patient diagnosed with traumatic dislocation of the tibialis posterior tendon as a result of twisting injury after falling from a^ ^1-meter jump height while playing basketball; the patient was clinically diagnosed primarily as a case of simple ankle sprain in the emergency department and treated conservatively with cold compression. The patient was seen in the clinic after five days with the same complaint, which was medial ankle pain without any improvement. Proper examination and imaging techniques lead us to the accurate diagnoses. The patient was managed surgically and had an excellent outcome postoperatively with a full range of motion of the ankle joint and full weight-bearing without any complaint.

Tibialis posterior dislocation should be one of the possible differential diagnoses while dealing with any ankle injury even with unremarkable X-rays. History and physical examination, if conducted correctly, are the keys to making an accurate diagnosis. Therefore, we recommend a proper history-taking and precise physical examination with a high index of suspicion for any possible diagnoses. Early surgical intervention for such cases is the preferable method of treatment to avoid further complications and promote early functional recovery.

## Introduction

The tibialis posterior tendon (TPT) has a significant role in the plantar flexion of the ankle and inversion of the foot. It is also an essential stabilizer of the medial longitudinal arch. TPT dysfunction can lead to acquired flatfoot deformity in adults. The origin of the TPT is from the lateral part of the posterior surface of the tibia, the medial part of the posterior surface of the fibula, and the interosseous membrane, with insertion in the plantar aspect of the navicular bone, the cuboid, the cuneiforms, and all the metatarsal bases except the first one. The TPT lies immediately posterior to the medial malleolus passing through a fibro-osseous tunnel where it changes direction acutely, like a bowstring. The tunnel consists of the retromalleolar groove in the distal tibia and the overlying flexor retinaculum. The retinaculum keeps the TPT in the groove, preventing its dislocation. The depth and width of the groove vary significantly. In a cadaveric study, Soler et al. reported groove variation in the width ranging from 6 to 15 mm and that in the depth ranging from 1.5 to 4 mm [1]. CT is a beneficial imaging tool to configure the size of the retromalleolar groove and helps in the preoperative planning of the case [2]. The TPT dislocates anteriorly to the medial malleolus, either subcutaneously or subperiosteally. In the acute stage, TPT dislocation presents with a moderate to severe pain with mild swelling in the medial aspect of the ankle and is frequently misdiagnosed as ankle sprain; this can delay appropriate surgical treatment [3]. Generally, surgical treatment is far more preferable and effective than conservative management according to most authors [2].

## Case presentation

**Figure 1:**
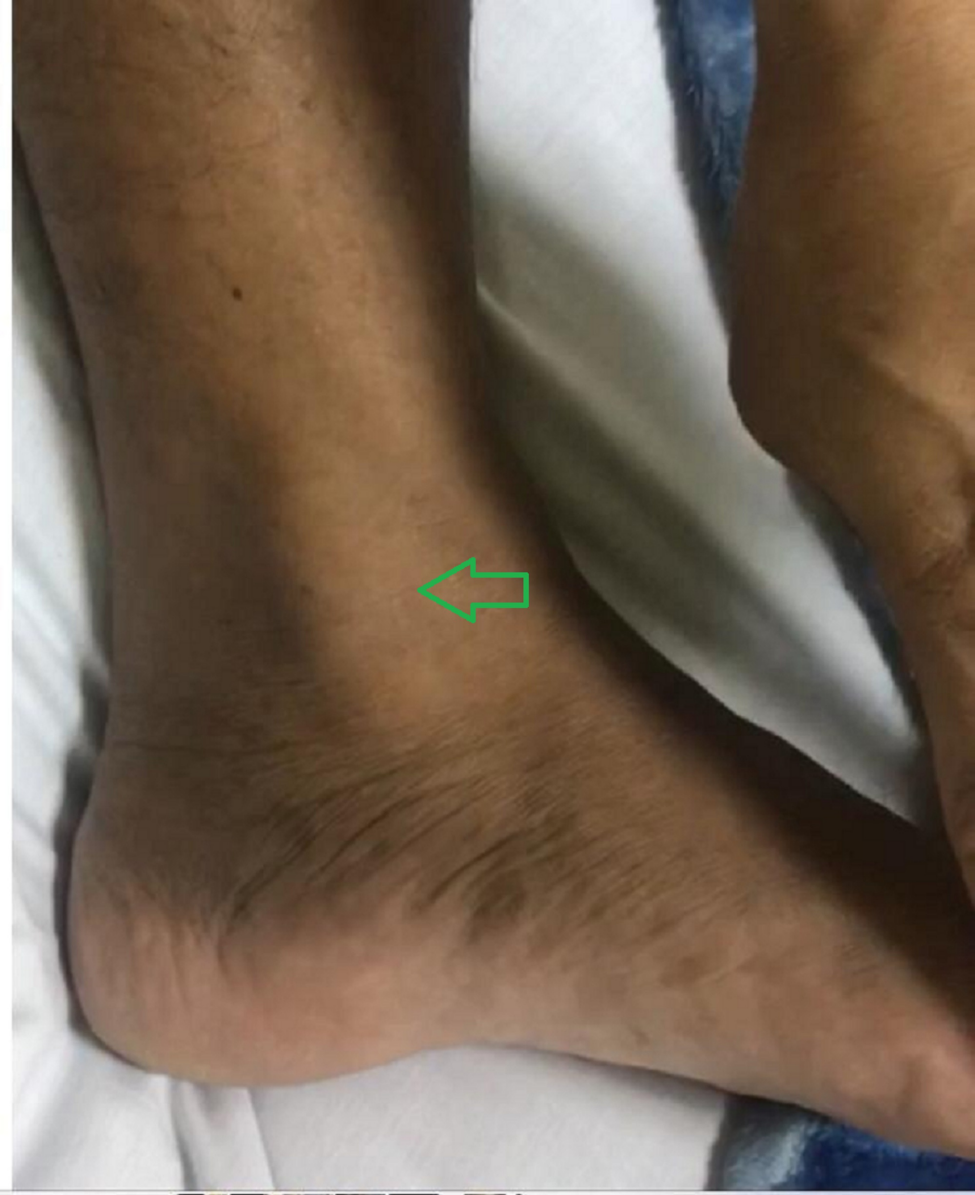
A photograph of the medial aspect of the left ankle showing a longitudinal cord-like structure over the medial malleolus (green arrow).

**Figure 2:**
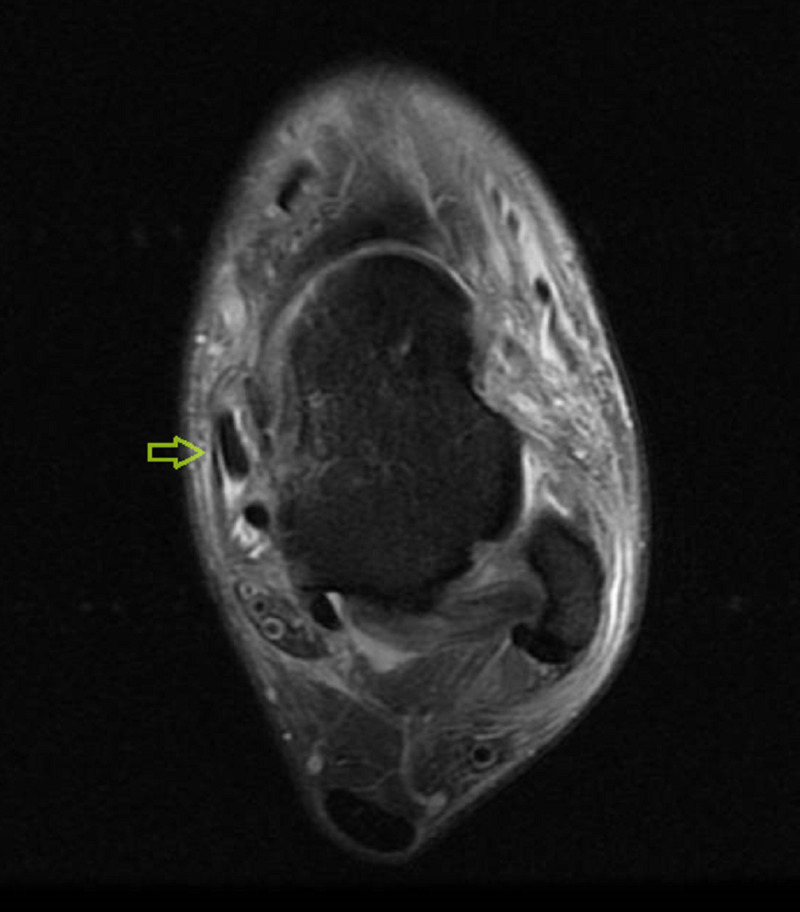
T2-weighted MRI (transverse plane) demonstrating an anterior subluxation of the tibialis posterior tendon over the medial malleolus (green arrow).

**Figure 3:**
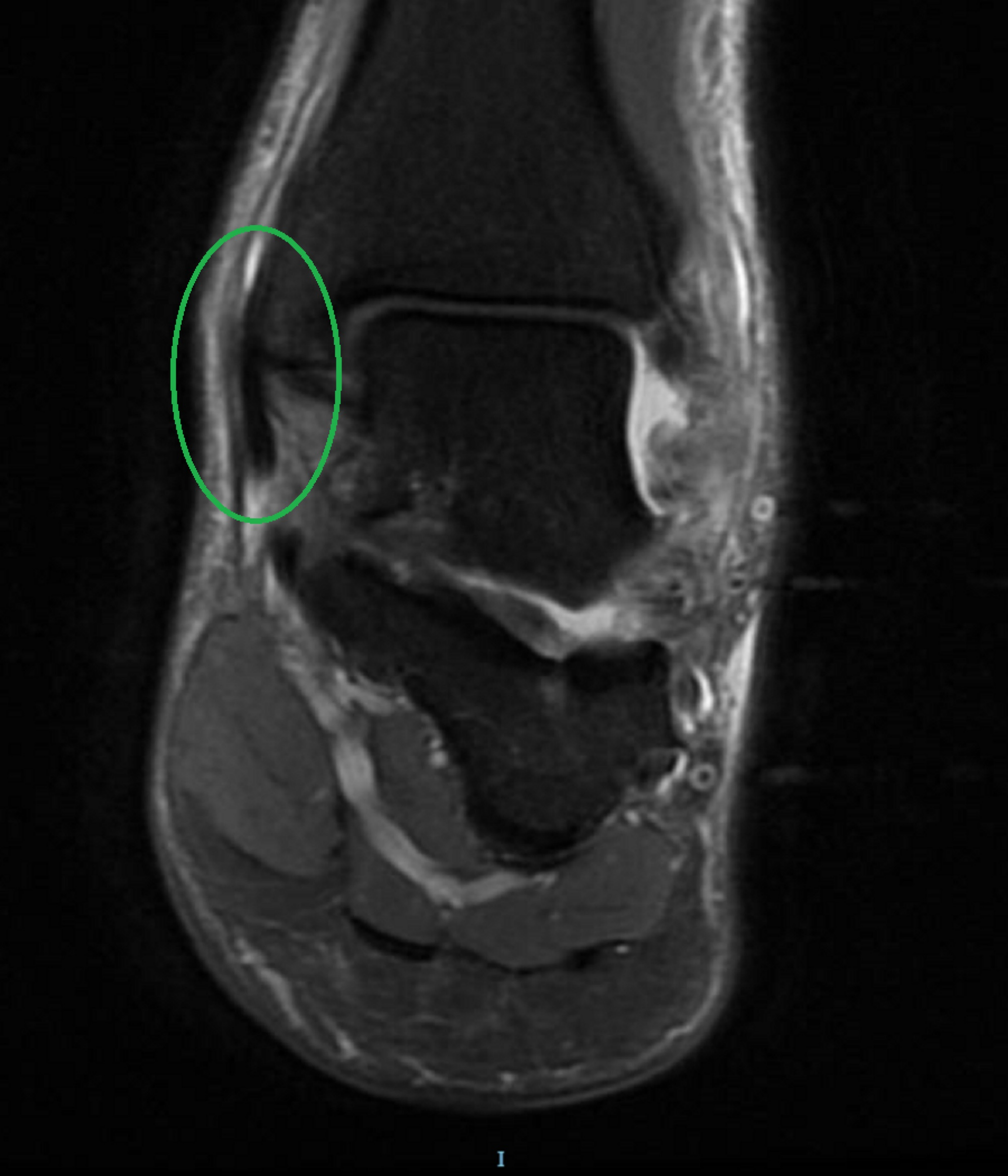
T2-weighted MRI (coronal plane) demonstrating an anterior subluxation of the posterior tibialis tendon over the medial malleolus with mild effusion (green circle), which should appear posterior to the medial malleolus.

**Figure 4:**
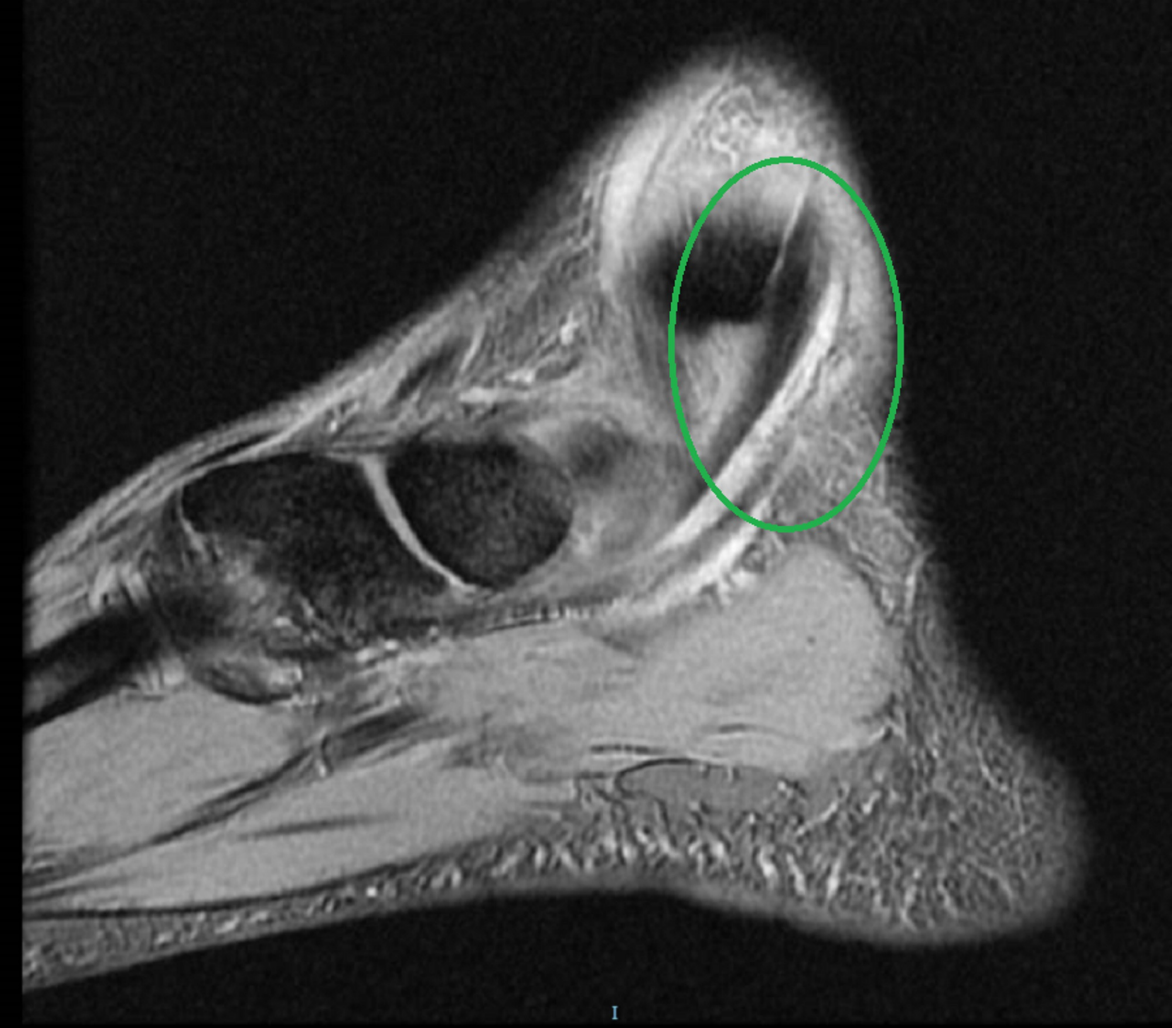
T2-weighted MRI (sagittal plane) demonstrating an anterior subluxation of the posterior tibialis tendon (green circle) over the medial malleolus with mild hypertrophy of the tibialis posterior tendon.

A 39-year-old male firefighter presented to the emergency department with left ankle pain and inability to bear weight after falling from a 1-meter jump height while playing basketball. He reported landing on the dorsum of his left foot with the ankle in the plantar flexion position. Clinically, there was moderate ankle swelling in the medial aspect with a limited range of motion of the ankle joint. His X-rays were unremarkable. He was diagnosed with an ankle sprain and was managed with rest, ice, elevation, compression, and analgesia. Five days later, he presented to the orthopedic clinic with the same complaints. On examination, he had localized swelling just anterior to the medial malleolus. Upon palpation, there was a slightly tender cord-like structure that moved back and forth with gentle plantar and dorsiflexion of the ankle (Figure 1; Video 1). Traumatic dislocation of the TPT was suspected, and an urgent MRI was performed. The MRI revealed a significant thickening of the TPT, tenosynovitis of the adjacent flexor digitorum longus, a possible tear of the flexor retinaculum, and a partial tear of the superficial deltoid ligament (Figures 2-4). The patient was offered surgery on the next elective list.

**Video 1:** A video tape showing the medial aspect of the left ankle, which shows a longitudinal cord-like structure moving over the medial malleolus with dorsi flexion of the ankle (unstable tibialis posterior tendon)

Operative procedure

**Figure 5:**
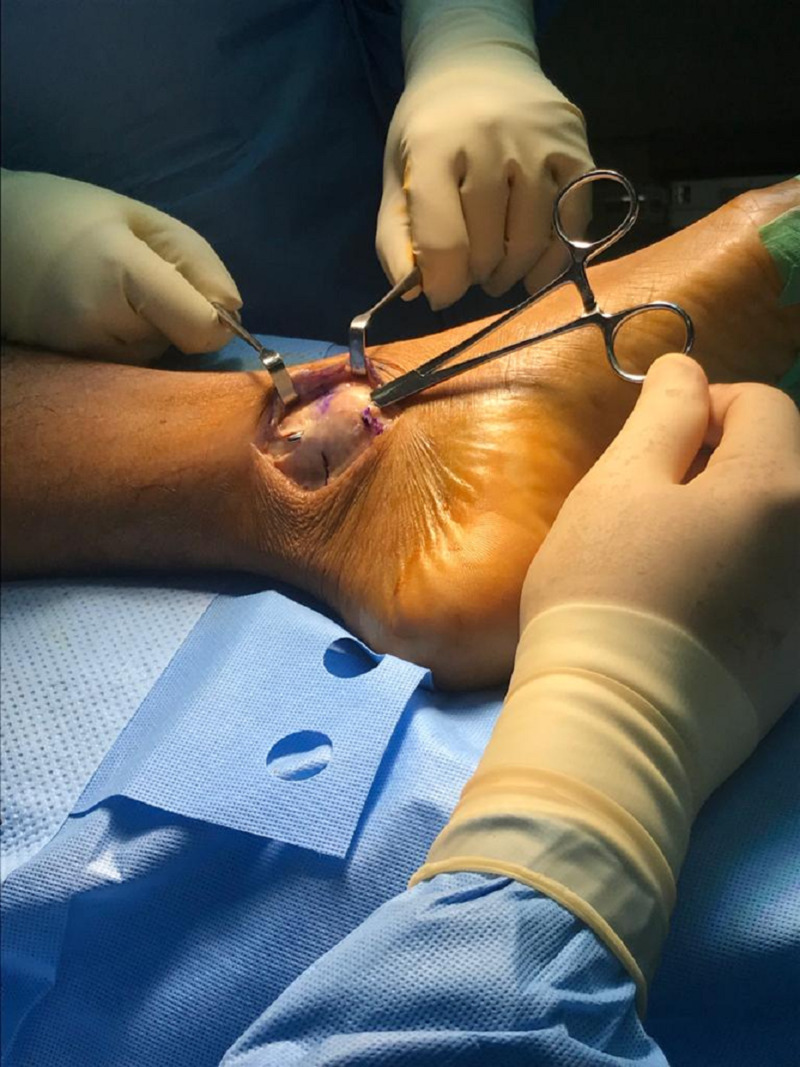
Intra-operative photo showing the 6-cm longitudinal incision along with the dorsal edge of the medial malleolus with intact flexor retinaculum.

**Figure 6:**
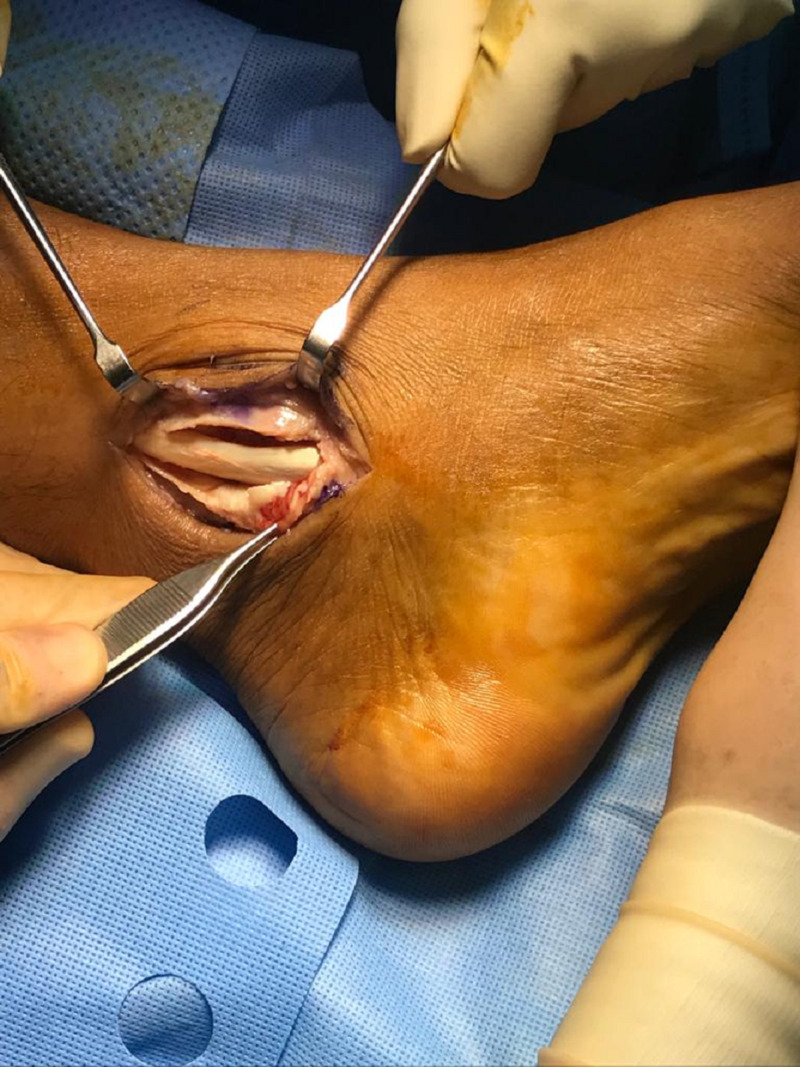
Intra-operative photo showing the dislocation of the intact tibialis posterior tendon with a shallow retromalleolar groove after incising the flexor retinaculum.

**Figure 7:**
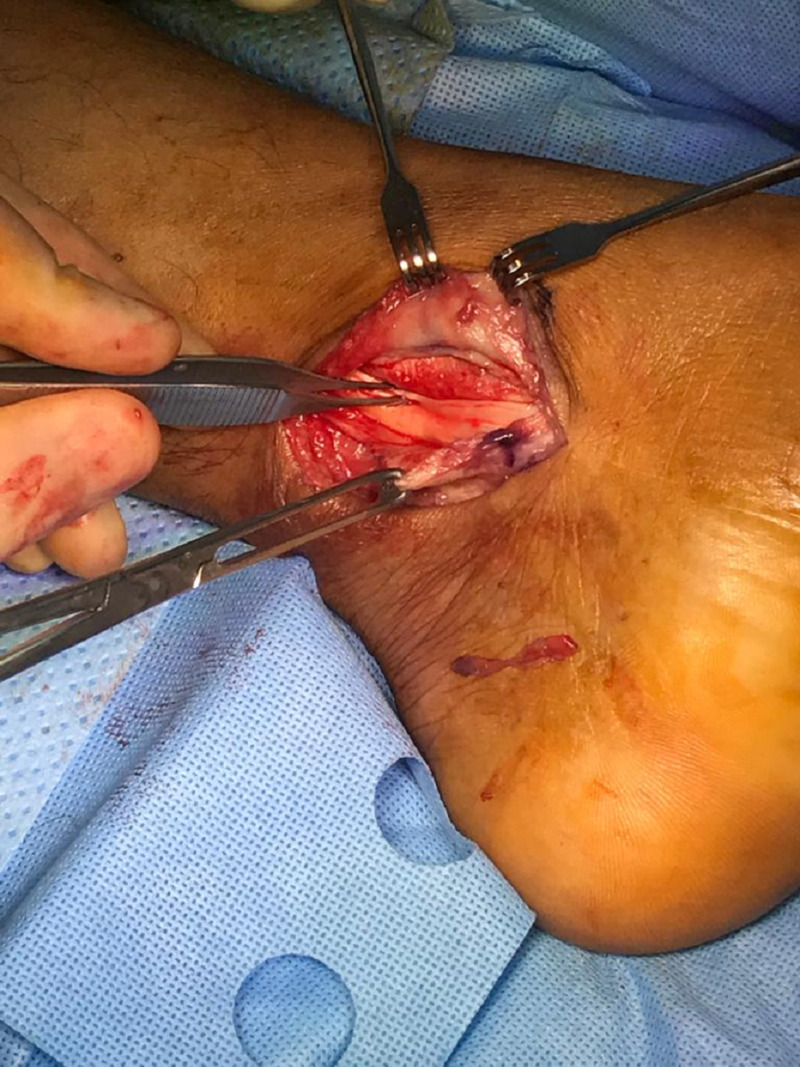
Intra-operative photo showing reduced tibialis posterior tendon to the retromalleolar groove.

**Figure 8:**
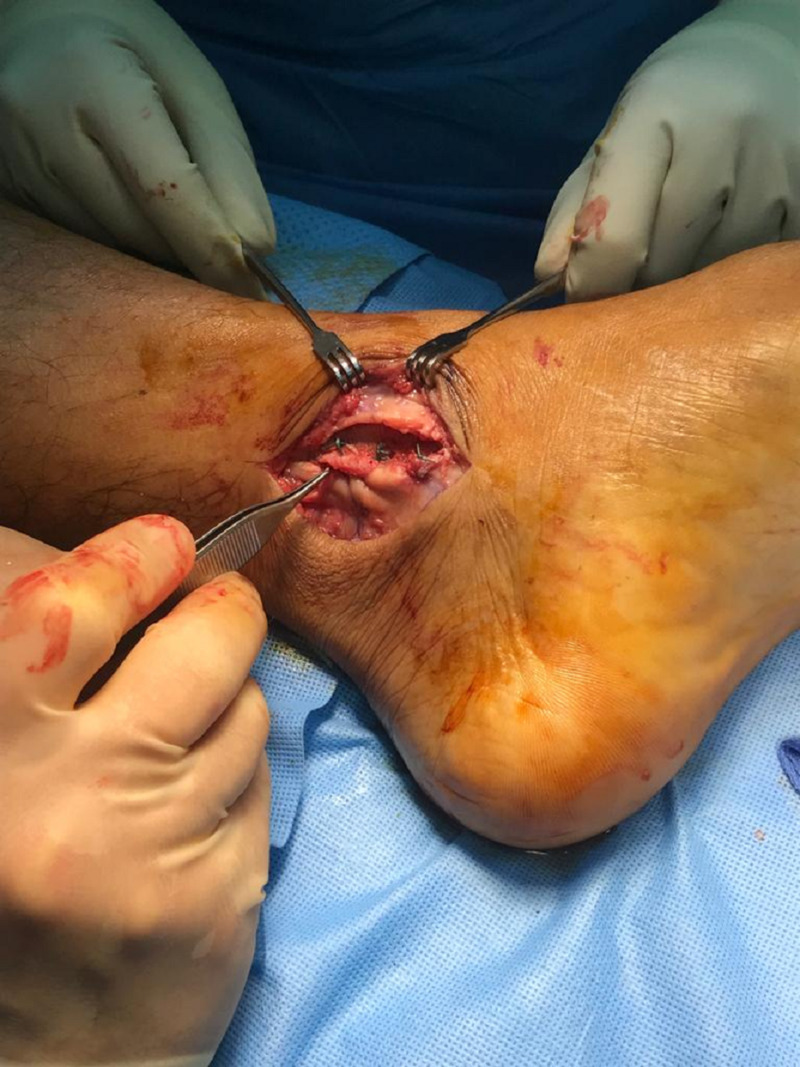
Intra-operative photo showing repairing of the flexor retinaculum.

The patient was placed in supine position, and to facilitate external rotation of the left leg, a bolster was placed under the contralateral hip. A 6-cm longitudinal incision was made along the anterior aspect of the medial malleolus (Figure 5). The retinaculum was found intact; however, its anterior continuity with the periosteum was avulsed as one layer from the underlying bare bone. After incising the retinaculum, the tendon was found subperiosteally anterior to the medial malleolus. Intra-operatively, the retromalleolar groove was found to be shallow compared with the size of the tendon (Figure 6), trials of relocation of the unstable tendon were attempted, and the posterior tibialis tendon was irreducible in the groove. Therefore, to deepen the groove, a longitudinal osteotomy at the edge of the groove was performed using a small, thin osteotome. The fibrocartilage surface of the groove floor was preserved while the shell of bone was levered as a flap with the osteotome to expose the underlying cancellous bone. A curette was used to remove a small amount of the cancellous bone to make a gutter. The flap was then over-reduced to make a concavity. This last step was performed by tapping on the side of a rounded bone tamp that was placed in the groove over a piece of gauze. The TPT was reduced in the groove and found to be stable (Figure 7), and the retinaculum was repaired with non-absorbable sutures (Figure 8). A couple of sutures were passed through the posterior edge of the medial malleolus. Care was taken to avoid over-tightening the retinaculum so that the tendon was freely mobile with a smooth gliding motion. A layered closure was performed, and a short back slab was applied.

Postoperative management

The patient was permitted to touch-down on the back slab using a pair of crutches for six weeks. After the back slab was removed, ankle motion was started and progressive weight-bearing was allowed. The patient had regular follow-up visits for 12 months.

Outcome

After three months, the patient had no pain or swelling in the ankle. He was able to bear full weight, and he had a full ankle range of motion. He was allowed to play sports after six months. At 12 months, he was asymptomatic and was back to his pre-injury level of activity.

## Discussion

Traumatic dislocation of the TPT is an uncommon injury. Approximately only 50 cases have been reported and have reviewed the TPT dislocation in the published literature [3]. The first case was reported by Martius in 1874 [3]. Many orthopedic surgeons are unfamiliar with this condition due to its rarity [2]. Accordingly, it is usually misdiagnosed as an ankle sprain. However, a high index of suspicion should be exercised to avoid delaying surgical intervention, which is the mainstay of management. If neglected, multiple complications can develop. These include chronicity and recurrence, rupture of the TPT leading to an adult-onset flatfoot, valgus deformity, and chronic pain. To avoid or minimize these problems, surgical intervention should be considered even in the acute setting [4]. Furthermore, a delay in the diagnosis and definitive management in athletes prolongs their recovery time and ability to return to sports [4]. Traumatic TPT dislocation is caused by different mechanisms of injury, including plantar flexion with inversion, falling in a varus position of the foot, from a recurrent forced inversion from twisting injuries, or as a result of a motor vehicle accident [4]. Dislocation in our patient was due to plantar flexion and inversion while he landed from a jump. In most of the reported cases, it was easy to palpate the tendon anterior to the medial malleolus. It is important to rule out bony injuries by standard radiographs. When suspected, an additional internal rotation (Mortise) view can help to find any bony avulsion from the insertion of the flexor retinaculum [4]. Ultrasonography is rarely used to diagnose the TPT dislocation, and there is only one literature has been reported the appearance and the integrity of the TPT [5].

CT scan is the radiological study of choice to detect hypoplasia of the retromalleolar groove, but it was not performed for our patient since it has limitations in evaluating the soft tissue injuries and it exposes the patient to high level of radiation [6]. MRI is the modality of choice to detect the dislocated tendon and associated soft tissue injuries and to assess the integrity of the flexor retinaculum, and therefore it was superior over the CT scan in this case [7]. Another modality that can be used to understand the dynamic motion of the TPT after the injury is ultrasonography but it is technically demanding [5].

There are two dislocation types reported in the literature: type I, flexor retinaculum tear leading to subcutaneous dislocation, and type II, periosteal avulsion of retinaculum leading to subperiosteal dislocation [8]. Our patient had type II dislocation.

One of the unusual traumatic injuries of the ankle fractures is the interposition of the TPT or flexor digitorum longus tendon which results in irreducible fracture-dislocation in the medial aspect of the ankle. The TPT is an important anatomical structure, which is the most common cause of interposition in irreducible ankle fracture dislocation due to the entrapment of the TPT in the tibiofibular and tibiotalar joints [9-10].​​​

Another unusual traumatic injury to the TPT is a full or partial rupture of the tendon associated with ankle bimalleolar or medial malleolar fracture; these kinds of injuries happen in case of pronation and external rotation ankle fracture causing the most tension to the TPT or in case of a direct trauma to the posterior aspect of the medial malleolus, which may cause a direct injury to the TPT [11-12].

Most authors have reported that surgical intervention is more effective than conservative management [2]. Ouzounian and Myerson reported the biggest series of this condition, featuring seven patients. All seven patients failed conservative management in the form of casting, bracing, and physical therapy, and eventually they all required surgical management. The standard surgical intervention is tendon reduction and flexor retinaculum repair. In the case of hypoplasia of the retromalleolar groove, some patients may need additional procedures such as groove deepening or bone grafting.

## Conclusions

TPT dislocation is a very rare condition and can be easily missed in acute settings. However, a detailed history and careful physical examination combined with appropriate imaging techniques can lead to a timely and accurate diagnosis. If TPT dislocation is suspected, the use of ultrasonography or MRI is indicated to confirm the clinical diagnosis. We recommend practicing a high index of suspicion when dealing with ankle injuries to avoid subsequent delay in diagnosing the case and to provide the appropriate management.
